# Bayesian Estimation of Marginal Quantiles with Missing Data in a Multivariate Regression Framework

**DOI:** 10.3390/e28020201

**Published:** 2026-02-10

**Authors:** Raúl Alejandro Morán-Vásquez, Mauricio A. Mazo-Lopera, Jose Antonio Escobar-Arias

**Affiliations:** 1 Instituto de Matemáticas, Universidad de Antioquia, Calle 67 No. 53-108, Medellín 050010, Colombia; 2 Departamento de Estadística, Universidad Nacional de Colombia, Carrera 65 No. 59A-110, Medellín 050034, Colombia; mamazol@unal.edu.co (M.A.M.-L.); joescobara@unal.edu.co (J.A.E.-A.)

**Keywords:** Bayesian analysis, monotone data augmentation algorithm, missing data, multivariate linear regression, quantile modeling

## Abstract

In this article, we propose and study a class of multivariate regression models that account for ignorable missing data in skewed, potentially heavy-tailed response vectors with positive components. It can be used to estimate the marginal quantiles of the response vectors based on a set of covariates, while considering the potential association among the components of the response vectors. We adopt an MCMC Bayesian approach to perform the posterior analysis via a monotone data augmentation algorithm for data imputation. The satisfactory performance of the posterior distributions and the handling of missing data in quantile estimation are verified through simulation studies. The procedures are illustrated using real children’s anthropometric data.

## 1. Introduction

### 1.1. Background and Motivation

Multivariate missing data are common in empirical studies. When missing values occur across multiple variables simultaneously, they create interconnected patterns that make statistical analysis difficult. Most statistical methods for handling multivariate missing data in response vectors within regression analysis are primarily developed under the assumption of multivariate symmetry. The multivariate normal/independent (NI) class of distributions has been employed in robust regression where the response vector contains missing values (Liu [1], Little [2]). This class offers important theoretical properties and includes heavy-tailed distributions where expectation-maximization algorithms can be effectively used for maximum likelihood estimation (Lange et al. [3], Lange and Sinsheimer [4]). However, a drawback of regression models that rely on multivariate NI distributions is that they do not account for the potential skewness, a typical feature when dealing with data of a response vector with positive components. The modeling of the association between a set of covariates and a continuous, positive response vector under skewness and outliers has received limited attention in statistical research and has primarily been conducted under the assumption of complete data. Some approaches for this situation involve the multivariate Box-Cox transformation (Gnanadesikan [5]), multivariate covariance generalized linear models (Bonat and Jørgensen [6]), and regression models based on multivariate asymmetric distributions with heavy tails and positive support (Marchant et al. [7], Morán-Vásquez et al. [8]).

Quantile regression (Koenker and Bassett [9]) provides a robust framework for modeling conditional distributional characteristics beyond the mean. However, extending this to multivariate settings remains challenging due to the interdependence of responses and the practical reality that data are rarely fully observed. This work introduces a new class of regression models built upon the multivariate log-normal/independent (LNI) distributions (Morán-Vásquez et al. [8]), with fully observed explanatory variables and a response vector that may contain missing values. Our models allow us to investigate the dependence of the marginal quantiles of the response vector on a set of covariates, while accounting for skewness and possible outliers. This allows easy interpretation of the regression coefficients, an advantage from a regression modeling viewpoint. Other parameters control the marginal relative dispersions and the correlations among the marginal responses. Additionally, the regression models we introduce are based on distributions with heavier (or lighter) tails than the classical multivariate log-normal distribution, including the multivariate log-*t*, log-slash, and log-power-exponential distributions (Morán-Vásquez et al. [8], Morán-Vásquez and Ferrari [10]), providing several alternatives for handling outliers in the response vector. We carry out estimation procedures from a Bayesian perspective, employing the equivalence between the multivariate LNI and NI distributions via logarithmic transformation (Morán-Vásquez et al. [8]). We employ a monotone data augmentation (MDA) algorithm [1,11,12] for multiple imputation of incomplete data, which enables the computation of the joint posterior distribution of the model parameters. Our simulation studies will assess the approach to approximate the joint posterior distributions and the marginal quantile estimation procedure. Additionally, we illustrate our methodology using real data on anthropometric measurements of children up to and including 4 years old.

### 1.2. Related Literature

Few works on multivariate quantile regression have been addressed in the literature compared to those on univariate quantile regression, and they primarily assume that complete data are available. Some approaches based on classical parametric and non-parametric methods can be found in Chakraborty [13], Wei [14], Hallin et al. [15], McKeague et al. [16], Petrella and Raponi [17], and Morán-Vásquez et al. [8]. Additionally, some works on multivariate quantile regression that use Bayesian approaches appear in Waldmann and Kneib [18], Bhattacharya and Ghosal [19], and Guggisberg [20]. Missing observations are common in datasets but have received little attention in quantile regression. Early approaches include imputation methods that generate values from conditional quantile functions (Yoon [21]), iterative multiple imputation procedures for quantile regression models with missing covariates (Wey et al. [22]), as well as smoothed empirical likelihood methods (Luo et al. [23]), fractional hot deck imputation, and both parametric and nonparametric fractional imputation (Shu et al. [24]) for handling missing response values.

While recent advances, such as functional-coefficient quantile regression for structured panel data (Yang et al. [25]) and neural network-based joint-output methods (Hao and Yang [26]), signal a growing interest in joint quantile modeling, these approaches typically assume complete response vectors and fail to directly address multivariate missingness. In applied domains ranging from energy systems to spatiotemporal monitoring (Li et al. [27]; Wang et al. [28]), principled missing data treatment and accounting for joint dependence have proven crucial for reliable inference. Furthermore, a Bayesian perspective suggests that recent strides in adaptive computational algorithms are driving the application of structured data augmentation techniques to posterior inference in complex regression models with unavailable multivariate responses (Tian et al. [29]). Environmental and spatial datasets frequently exhibit missing values as a consequence of data collection limitations, such as sensor failures or irregular monitoring schemes (Bianco et al. [30]). Survey data commonly contain missing observations due to item nonresponse or reporting errors, as some respondents decline to answer certain questions or provide inaccurate information (Xu et al. [31]). In medical studies, data obtained from heterogeneous assessment instruments may be systematically missing for subsets of participants, reflecting study design constraints or patient-specific factors (Xue et al. [32]). In these settings, multivariate quantile regression offers a robust framework for analyzing correlated variables subject to patterns of missingness.

### 1.3. Organization

We structure the article as follows. Section 2 briefly outlines the multivariate LNI distributions. Section 3 describes the Bayesian estimation approach of the multivariate LNI linear regression models via the MDA algorithm. In Section 4, we report the findings from simulation studies. Section 5 focuses on applications to children’s anthropometric data. The paper concludes in Section 6, where we provide the discussion and conclusions.

## 2. The Class of Multivariate LNI Distributions

Multivariate models provide a more realistic representation of complex phenomena by analyzing random variables jointly rather than in isolation, thereby improving estimation precision and enhancing predictive performance. Moreover, such models enable the identification and interpretation of complex relationships and interactions that cannot be captured by single-variable approaches (Kramer [33]). The class of multivariate LNI distributions (Morán-Vásquez et al. [8]) encompasses a broad family of models that allow for the straightforward modeling of marginal quantiles, a key feature in quantile regression.

Throughout this paper, we employ bold lowercase Greek letters for vectors and bold uppercase Greek letters for matrices. The entries of vectors and matrices are represented using the corresponding Greek letter in regular font. A similar notation is employed for random vectors and matrices, but written in uppercase Roman letters. If $\theta \in R_{+}^{p}$, we define $\log\left(\theta \right)={\left(\log\left(\theta_{1}\right),\ldots ,\log\left(\theta_{p}\right)\right)}^{\prime }$. If $\theta \in R^{p}$, we define $\exp\left(\theta \right)={\left(\exp\left(\theta_{1}\right),\ldots ,\exp\left(\theta_{p}\right)\right)}^{\prime }$. For a square matrix Σ, we denote its determinant and trace by det(Σ) and tr(Σ), respectively. Moreover, we denote etr(Σ)=exp(tr(Σ)). We use the notation Σ>0 to represent that the square matrix Σ is positive definite.

We define a multivariate log-normal random vector $Y\in R_{+}^{p}$ with parameters $\mu \in R_{+}^{p}$ and Ψ(p×p)>0, by the probability density function (PDF):

$${\mathrm{LN}}_{p}\left(y\;|\;\mu ,\Psi \right)=\phi_{p}\left(\log\left(y\right)\;|\;\log\left(\mu \right),\Psi \right)\prod_{k=1}^{p}\frac{1}{y_{k}},$$

where $\phi_{p}\left(x\;|\;\xi ,\Sigma \right)={\left(2\pi \right)}^{-p/2}\det{\left(\Sigma \right)}^{-1/2}\exp\left(-\delta^{2}\left(x;\xi ,\Sigma \right)/2\right)$ denotes the well-known density of the multivariate normal distribution $X∼N_{p}\left(\xi ,\Sigma \right)$. The quantity $\delta^{2}\left(x;\xi ,\Sigma \right)={\left(x-\xi \right)}^{\prime }\Sigma^{-1}\left(x-\xi \right)$ represents the squared Mahalanobis distance between x and $\xi \in R^{p}$ with respect to Σ(p×p)>0.

The multivariate LNI random vector $Y\in R_{+}^{p}$ with parameters $\mu \in R_{+}^{p}$ and Ψ(p×p)>0 is defined via the stochastic representation:

(1) $$Y|w∼{\mathrm{LN}}_{p}\left(\mu ,\Psi /w\right),\;w∼H\left(w\;|\;\nu \right),$$

where H(·|ν) is the CDF of w>0, and $\nu \in R^{q}$ is a vector of additional parameters associated with *H*. The parameters $\mu \in R_{+}^{p}$ and Ψ(p×p)>0 are interpreted as the median vector and the dispersion matrix, respectively. Any random vector $Y\in R_{+}^{p}$ that satisfies stochastic representation (1) is said to follow a multivariate LNI distribution, denoted by $Y∼{\mathrm{LNI}}_{p}\left(\mu ,\Psi ,H\right)$.

If h(·|ν) denotes the PDF of w>0, then the PDF of $Y∼{\mathrm{LNI}}_{p}\left(\mu ,\Psi ,H\right)$ is given by

(2) $${\mathrm{LNI}}_{p}\left(y\;|\;\mu ,\Psi ,\nu \right)=\int_{0}^{\infty }{\mathrm{LN}}_{p}\left(y\;|\;\mu ,\Psi /w\right)h\left(w\;|\;\nu \right)\;dw.$$

The random variable *w* uniquely determines each family within the class of multivariate LNI distributions. Consequently, provided that *w* has a degenerate distribution at 1, then in (2) we obtain the PDF of $Y∼{\mathrm{LN}}_{p}\left(\mu ,\Psi \right)$. If w∼Gamma(ν/2,ν/2), with PDF

(3) $$h\left(w|\nu \right)=\frac{{\left(\nu /2\right)}^{\nu /2}}{\Gamma (\nu /2)}w^{\nu /2-1}\exp\left(-\nu w/2\right),\;\nu >0,\;w>0,$$

we obtain the density of the multivariate log-*t* distribution with parameters $\mu \in R_{+}^{p}$, Ψ(p×p)>0, and ν>0. The additional parameter ν is called the degrees of freedom parameter. When w∼Beta(ν,1), ν>0, with PDF

(4) $$h\left(w|\nu \right)=\nu w^{\nu -1},\;\nu >0,\;0<w<1,$$

we obtain the PDF of the multivariate log-slash distribution with parameters $\mu \in R_{+}^{p}$, Ψ(p×p)>0, and ν>0. The additional parameter ν is referred to as the tail parameter. The multivariate log-*t* and log-slash distributions are suitable for handling outliers, as they exhibit heavier tails compared to the multivariate log-normal distribution for small values of ν. Moreover, one recovers the multivariate log-normal distribution by letting ν tend toward infinity in either the log-*t* or log-slash models. Other special cases within the multivariate LNI class include the multivariate log-contaminated normal distribution, the multivariate log-Pearson type VII distribution, and the multivariate log-Laplace distribution, among many others.

Log-elliptical distributions (Morán-Vásquez and Ferrari [10]) form a larger class containing the LNI class, which connects directly to the NI distributions (Lange and Sinsheimer [4]) via the log-transformation. In fact, if $Y∼{\mathrm{LNI}}_{p}\left(\mu ,\Psi ,H\right)$, then $T=\log\left(Y\right)∼{\mathrm{NI}}_{p}\left(\eta ,\Psi ,H\right)$, where $\eta =\log\left(\mu \right)\in R^{p}$ is a location vector, and Ψ(p×p)>0 is a dispersion matrix. The above allows us to express the marginal quantiles of the multivariate LNI random vectors in terms of the quantiles of standard univariate NI random variables. So, if $Y∼{\mathrm{LNI}}_{p}\left(\mu ,\Psi ,H\right)$, then the α-quantile $y_{k,\alpha }$ of $Y_{k}$, α∈(0,1), satisfies

(5) $$y_{k,\alpha }=\mu_{k}\exp\left(\sqrt{\psi_{kk}}q_{\alpha }\right),$$

for k=1,…,p, where $q_{\alpha }$ is the α-quantile of $Z∼{\mathrm{NI}}_{1}\left(0,1,H\right)$. Observe that $y_{k,1/2}=\mu_{k}$, which implies that $\mu_{k}$ represents the median of $Y_{k}$. Furthermore, the parameter $\psi_{kk}$ impacts the relative dispersion of $Y_{k}$, as seen in the relationship:

$$\mathrm{CV}Y_{k}=1.5\sinh\left(\sqrt{\psi_{kk}}q_{3/4}\right),$$

for k=1,…,p, where ${\mathrm{CV}}_{Y_{k}}$ represents the quantile-based coefficient of variation for $Y_{k}$, as defined by Rigby and Stasinopoulos [34]:

$${CV}_{Y_{k}}=\frac{3}{4}\frac{\left(y_{k,3/4}-y_{k,1/4}\right)}{y_{k,1/2}},$$

for k=1,…,p. The parameter $\psi_{jk}$ controls the association between the random variables $Y_{j}$ and $Y_{k}$.

Additional results regarding the multivariate LNI distributions are available in Morán-Vásquez et al. [8].

## 3. Joint Estimation of Marginal Quantiles via the Class of Multivariate LNI Linear Regression Models with Missing Data

### 3.1. Multivariate LNI Linear Regression with Missing Values in Response Variables

The multivariate LNI distributions can be naturally embedded within linear regression frameworks (Morán-Vásquez et al. [8]). This formulation is particularly powerful because it enables the interpretation of regression coefficients in terms of marginal quantiles, while simultaneously accounting for the association among response variables, multivariate skewness, and potential outliers. These features motivate the development of multivariate LNI linear regression models capable of handling missing values in the response vector. For such models, a Bayesian approach using the MDA algorithm (Liu [1]) is highly appropriate.

It is assumed that $Y_{1},\ldots ,Y_{n}$ are independent random vectors representing measurements of $Y\in R_{+}^{p}$ for *n* individuals. For i=1,…,n, $Y_{i}={\left(Y_{i1},\ldots ,Y_{ip}\right)}^{\prime }$ has possibly correlated components. Let $x_{i}={\left(x_{i1},\ldots ,x_{ir}\right)}^{\prime }$ be a fixed vector containing the values of the *i*-th individual for the explanatory variables $x_{1},\ldots ,x_{r}$. The class of multivariate LNI linear regression models is given by

(6) $$Y_{i}\left|B,\Psi ,X,w\overset{\mathrm{ind}}{∼}{\mathrm{LN}}_{p}\left(\exp\left(B^{\prime }x_{i}\right),\Psi /w_{i}\right),\;w_{i}\right|\nu \overset{i.i.d}{∼}H\left(w\;|\;\nu \right),$$

for i=1,…,n, where $X={\left(x_{1},\ldots ,x_{n}\right)}^{\prime }$ is the model matrix, $w={\left(w_{1},\ldots ,w_{n}\right)}^{\prime }$ is a vector of weights, Ψ(p×p)>0 is the dispersion matrix, $\nu \in R^{q}$ is a vector of additional parameters induced by *H*, and $B={\left(\beta_{jk}\right)}_{r\times p}$ is the matrix of regression coefficients, with $\beta_{jk}$ corresponding to $x_{ij}$, with $x_{i1}=1$, i=1,…,n, j=1,…,r.

We assume that the explanatory variables $x_{1},\ldots ,x_{r}$ are fully observed, while the observations of the response variables $Y_{1},\ldots ,Y_{p}$ can be arranged in a monotone missing data pattern; namely, for k=2,…,p, $Y_{k}$ is at least as observed as $Y_{k-1}$. Let $Y={\left(y_{ik}\right)}_{n\times p}$ be a matrix consisting of all observations from $Y_{1},\ldots ,Y_{p}$. Following Liu [1], the dataset [Y,X], comprising complete covariates and responses with a monotone missing pattern, is structured as:

(7) $$\left[Y_{\mathrm{MP}},X\right]=\left\{\left[y_{ik}^{\left(k\right)},\ldots ,y_{ip}^{\left(k\right)},x_{i1}^{\left(k\right)},\ldots ,x_{ir}^{\left(k\right)}\right]:k=1,\ldots ,p,i=1,\ldots ,n_{k}\right\},$$

where $\sum_{k=1}^{p}n_{k}=n$. Here, (k) acts as the index for the *p* available patterns. We recover a complete dataset from (7) when $n_{1}=n$.

We assume an ignorable missing data mechanism. Let $w=\{w_{i}^{\left(k\right)},k=1,\ldots ,p,i=1,\ldots ,n_{k}\}$ be the weights corresponding to (7). So, the class of linear regression model (6) associated with (7) is defined as

(8) $$Y_{i[k:p]}^{\left(k\right)}|B,\Psi ,X,w\overset{\mathrm{ind}}{∼}{\mathrm{LN}}_{p-k+1}\left(\exp\left(B^{(k)\prime }x_{i}^{\left(k\right)}\right),\Psi^{\left(k\right)}/w_{i}^{\left(k\right)}\right),\;w_{i}^{\left(k\right)}\overset{i.i.d}{∼}H\left(w\;|\;\nu \right),$$

for k=1,…,p, $i=1,\ldots ,n_{k}$, where $Y_{i[k:p]}^{\left(k\right)}={\left(Y_{ik}^{\left(k\right)},\ldots ,Y_{ip}^{\left(k\right)}\right)}^{\prime }$ is a random vector containing the responses of the *i*-th individual for $Y_{k},\ldots ,Y_{p}$ in the *k*-th pattern, $x_{i}^{\left(k\right)}={\left(x_{i1}^{\left(k\right)},\ldots ,x_{ir}^{\left(k\right)}\right)}^{\prime }$ is a fixed vector with the values of the *i*-th individual for $x_{1},\ldots ,x_{r}$ in the *k*-th pattern, $B^{\left(k\right)}$ denotes the r×(p−k+1) matrix consisting of the last p−k+1 columns of *B*, and $\Psi^{\left(k\right)}$ represents the trailing (p−k+1)×(p−k+1) principal submatrix of Ψ. Each family of (8) is characterized by the CDF *H*, providing a variety of linear regression models to analyze the dependence of the response vector on a set of predictors, while accounting for skewness, potential outliers, and missing data. Thus, we recover the log-normal case from (8) when *H* is the CDF of a degenerate distribution at w=1. The log-*t* and log-slash cases are derived from (8) when *H* is the CDF of a random variable *W* with PDFs given in (3) and (4), respectively. Other notable special cases within this class are the log-contaminated-normal, log-Pearson type VII, and log-Laplace models.

From Equation (13) of Morán-Vásquez et al. [8], we have that (8) is equivalent to

(9) $$\log\left(Y_{i[k:p]}^{\left(k\right)}\right)|B,\Psi ,X,w\overset{\mathrm{ind}}{∼}N_{p-k+1}\left(B^{(k)\prime }x_{i}^{\left(k\right)},\Psi^{\left(k\right)}/w_{i}^{\left(k\right)}\right),\;w_{i}^{\left(k\right)}\overset{i.i.d}{∼}H\left(w\;|\;\nu \right),$$

for k=1,…,p, $i=1,\ldots ,n_{k}$, which corresponds to a multivariate NI linear regression model (Lange and Sinsheimer [4]) with log-transformed response vector sorted into a monotone pattern with fully observed explanatory variables (Liu [1]). In this way, the Bayesian estimators of the parameters of (8) can be obtained through the Bayesian estimation of (9).

### 3.2. Bayesian Estimation via the MDA Algorithm

Following Liu [1], we assume that B, Ψ, and ν are independent a priori, and we propose an inverse Wishart distribution with parameter A(p×p)>0 and m+1 degrees of freedom as the prior for B and Ψ, that is

(10) $$P\left(B,\Psi \right)\propto \det{\left(\Psi \right)}^{-(m+1)/2}\mathrm{etr}\left(-\Psi^{-1}A/2\right).$$

The prior distribution for the vector of additional parameters depends on the specific distributional family under consideration. Thus, for the log-*t* case, we propose $P\left(\nu \right)\propto \nu^{-2}$ as the prior for the degrees of freedom parameter (Liu [12]). For the log-slash case, we suggest the conjugate prior ν∼Gamma(a,b), b≪a, for the tail parameter (Liu [1]).

To sample the parameters from their posterior distributions, we implement the MDA algorithm, which we outline in the following cases:

1. If the additional parameters ν and the weights w are known, and the data set has a monotone pattern, then the posterior simulation of (B,Ψ) can be performed using the following relationship:
  (11) $$P\left(B,\Psi |Y_{\mathrm{MP}},X,w\right)=P\left(\Psi |Y_{\mathrm{MP}},X,w\right)P\left(B|\Psi ,Y_{\mathrm{MP}},X,w\right),$$
  where samples from $P(\Psi |Y_{\mathrm{MP}},X,w)$ and $P(B|\Psi ,Y_{\mathrm{MP}},X,w)$ can be obtained using Theorem 1 and its corollaries from Liu [1]. In this case, MDA is non-iterative.
2. If there are missing values that destroy the monotone pattern, we construct a monotone pattern as $Y_{\mathrm{MP}}=\left[Y_{\mathrm{MP},\mathrm{mis}},Y_{\mathrm{obs}}\right]$, where $Y_{\mathrm{MP},\mathrm{mis}}$ contains the missing values needed to create the monotone pattern, and $Y_{\mathrm{obs}}$ consists of the observed values. In this case, it is sufficient to fill in the missing values $Y_{\mathrm{MP},\mathrm{mis}}$ with a sample from $P(Y_{\mathrm{MP},\mathrm{mis}}|B,\Psi ,Y_{\mathrm{obs}},X,w)$, which is a multivariate log-normal distribution, and then simulate (B,Ψ) from (11).
3. If the additional parameters ν are known and the weights w are unknown, it is sufficient to impute w with a draw from $P(w|B,\Psi ,Y_{\mathrm{obs}},X,\nu )$ by using the expression given in Liu [1] (Equation (7)), and then apply Case 2.
4. If the weights w and the additional parameters ν are unknown, we use the expectation maximization (EM) algorithm (Dempster et al. [35]) version of MDA. This consists of using Case 3 to impute $\left(Y_{\mathrm{MP},\mathrm{mis}},w\right)$ with the current values of (B,Ψ,ν), and then simulating (B,Ψ) using (11) and drawing ν from P(ν|w). Another method employs the expectation/conditional maximization (ECME) algorithm (Liu and Rubin [36]) version of MDA; see Liu [12].

Sampling of the additional parameters vector ν depends on the family considered in (8) and the prior specified for it. For the log-*t* case, we draw the degrees of freedom ν from the posterior provided in Liu [12] (Equation (11)). For the log-slash case, we draw the tail parameter ν from the posterior given in Liu [1] (Section 3.4). In both the simulation studies and the real data application presented in Section 4 and Section 5 of this article, we adopt Case 4 as described previously, where w and ν are unknown. The implementation of the MDA algorithm and the updating scheme for the model components depend on the specific distribution family considered. Accordingly, the steps for the log-*t* and log-slash cases are presented in Algorithms 1 and 2, respectively. For the detailed expressions of the posterior distributions involved in these algorithms, we refer the reader to Liu [1] (Section 3). To select initial values for B and Ψ, we first impute the missing responses (without covariates) using the approach presented in Schafer [37] (Section 6.5). We then fit a Bayesian multivariate normal linear regression model to this completed dataset and use the resulting estimates as the starting values for B and Ψ. Following Liu [12] (Section 2.3), we initialized ν=7 for the log-*t* and log-slash models.
**Algorithm 1** log-*t* family: ECME version of MDAStep 1: Draw the degrees of freedom parameter ν from $P(\nu |Y_{\mathrm{obs}},B,\Psi )$.
Step 2: Sample the weights w from $P(w|Y_{\mathrm{obs}},X,B,\Psi ,\nu )$.Step 3: Impute the missing response values that disrupt the monotone pattern from $P(Y_{\mathrm{MP},\mathrm{mis}}|Y_{\mathrm{obs}},X,B,\Psi ,w)$.Step 4: Update Ψ and B using (11).

**Algorithm 2** log-slash family: EM version of MDAStep 1: Sample the weights w from $P(w|Y_{\mathrm{obs}},X,B,\Psi ,\nu )$.Step 2: Impute the missing response values that disrupt the monotone pattern from $P(Y_{\mathrm{MP},\mathrm{mis}}|B,\Psi ,Y_{\mathrm{obs}},X,w)$.Step 3: Update B and Ψ using (11).Step 4: Update ν from $P(\nu |Y_{\mathrm{obs}},B,\Psi )$.

We compute the medians of each posterior sample to obtain the estimates $\hat{B}={\left({\hat{\beta }}_{jk}\right)}_{r\times p}$, $\hat{\Psi }={\left({\hat{\psi }}_{jk}\right)}_{p\times p}$ and $\hat{\nu }={\left({\hat{\nu }}_{1},\ldots ,{\hat{\nu }}_{q}\right)}^{\prime }$. So, from (5) and (6), we have that the α-quantile $y_{k,\alpha }$ of $Y_{k}$, α∈(0,1), is estimated as

(12) $${\hat{y}}_{k,\alpha }=\exp\left(\sum_{j=1}^{r}{\hat{\beta }}_{jk}x_{j}+\sqrt{{\hat{\sigma }}_{kk}}{\hat{q}}_{\alpha }\right),$$

for k=1,…,p. Here, ${\hat{q}}_{\alpha }$ represents the estimated α-quantile corresponding to the standard univariate normal/independent random variable $Z∼{\mathrm{NI}}_{1}\left(0,1,H\right)$, with $\nu =\hat{\nu }$. We compute ${\hat{q}}_{\alpha }$ for the standard *t* distribution using the algorithm provided by Hill [38], available in the R function qt. For the standard slash distribution, we first employ Monte Carlo integration to approximate its CDF (Morán-Vásquez and Ferrari [39] (Section 2)), and then we use root-finding algorithms to find ${\hat{q}}_{\alpha }$ (for example, the uniroot function in R). Note that if we plug α=0.5 into (12), then ${\hat{q}}_{\alpha }=0$. Consequently, the median is determined solely by the covariates via the exponential function, namely ${\hat{y}}_{k,\alpha }=\exp\left(\sum_{j=1}^{r}{\hat{\beta }}_{jk}x_{j}\right)$, k=1,…,p. From (12), we observe that increasing $x_{j}$ by one unit, holding the other covariates constant, results in a multiplicative change in ${\hat{y}}_{k,\alpha }$ by a factor of $\exp({\hat{\beta }}_{jk})$. Observe that, for a fixed *k*, the exponential impact of $x_{j}$ remains invariant across all α∈(0,1), thereby ensuring that the quantile curves do not cross.

## 4. Simulation Studies

We conducted simulation studies with the aim of assessing the approximation of the posterior distributions and the estimation parameter approach described in Section 3. The efficacy of the marginal quantile estimation technique was benchmarked through extensive Monte Carlo simulations. We consider the following multivariate LNI linear regression models:

$$Y_{i}\left|B,\Psi ,X,w\overset{\mathrm{ind}}{∼}{\mathrm{LN}}_{3}\left(\exp\left(B^{\prime }x_{i}\right),\Psi /w_{i}\right),\;w_{i}\right|\nu \overset{i.i.d}{∼}H\left(w\;|\;\nu \right),$$

for i=1,…,n, with fully observed explanatory variables $x_{2},x_{3},x_{4}$, and incomplete monotone response variables as given by (7). The simulations were carried out for the log-*t* and log-slash families, with the true parameters, $B={\left(\beta_{jk}\right)}_{4\times 3}$, $\Psi ={\left(\psi_{jk}\right)}_{3\times 3}$, and ν, obtained by fitting each regression model to the data set described in Section 5. We consider the arm circumference ($Y_{1}$), weight ($Y_{2}$), and length ($Y_{3}$) as response variables, and age ($x_{2}$), gender ($x_{3}$), and breastfeeding duration ($x_{4}$) as explanatory variables. We generated 1000 Monte Carlo samples for each family, varying the sample size across the set n∈{50,100,150}. We specifically chose these sample sizes to evaluate the asymptotic behavior of the posterior distributions and to guarantee the computational feasibility of the simulation. We simulated the covariates by drawing independent samples from distinct distributions. Specifically, $x_{2}$ and $x_{4}$ follow a gamma distribution with parameters estimated from the real data, while $x_{3}$ follows a Bernoulli distribution with a success probability of 0.61. These values remained fixed during the simulations. In each iteration, we generated 10,000 samples from the posterior distributions of the parameters and discarded the first 1000 samples during the burn-in period. We choose samples $y_{1},\ldots ,y_{n}$ such that $\sum_{i=1}^{n}\delta^{2}\left(\log\left(y_{i}\right);B^{\prime }x_{i},\Psi \right)>k_{n}$, with $k_{n}$ sufficiently large, in order to generate outliers similar to those in the real data. For each sample, we introduced missing values according to the proportion of missing values in each response variable from the real data set. The generation of missing entries was restricted to the non-outlying data, following the missing patterns from the real data set, namely: (obs, obs, obs), (mis, obs, obs), (obs, mis, obs), (obs, obs, mis), (mis, mis, obs), (mis, obs, mis), (obs, mis, mis), where “mis”denotes a missing value, and “obs”denotes an observed value. Following Liu [12], we adopt a non-informative prior for B and Ψ by setting A=0 and m=p in (10). Regarding ν, as previously mentioned, we assume $P\left(\nu \right)\propto \nu^{-2}$ for the degrees of freedom in the log-*t* family, and ν∼Gamma(6,2) for the tail parameter in the log-slash family.

**Figure A1:**
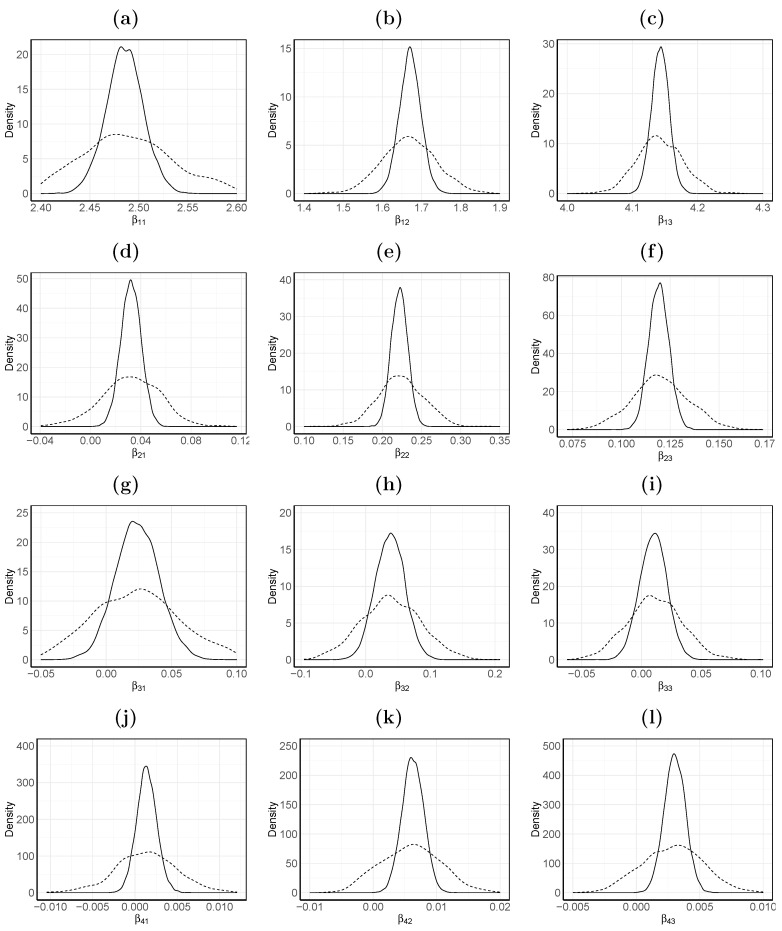
Comparison of the estimated posterior distribution (dashed line) of each regression coefficient with the true posterior distribution (solid line) for a sample size of n=50; log-*t* family.

**Figure A2:**
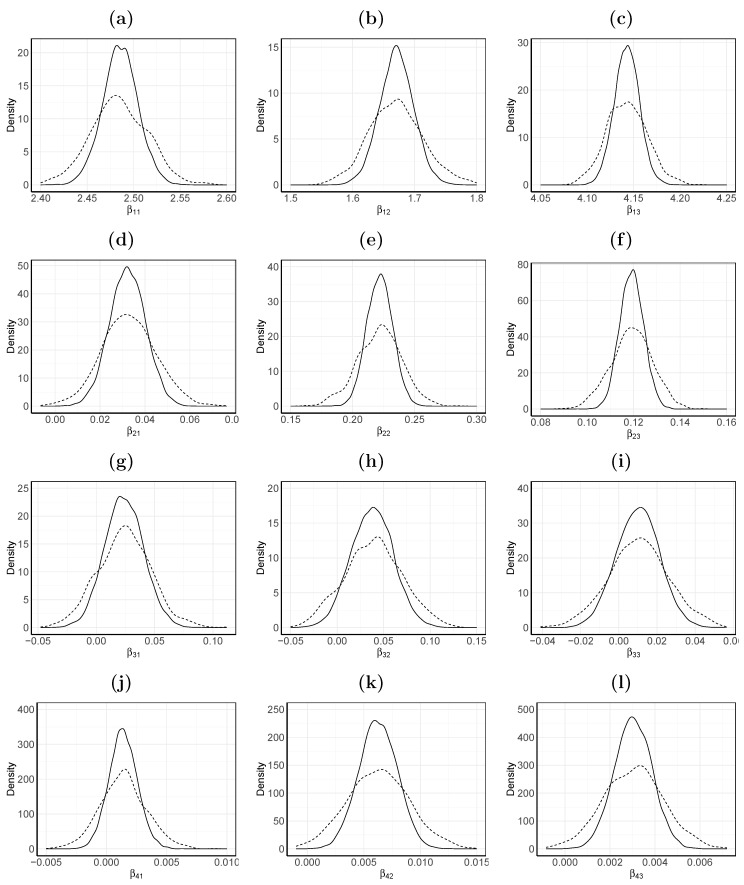
Comparison of the estimated posterior distribution (dashed line) of each regression coefficient with the true posterior distribution (solid line) for a sample size of n=100; log-*t* family.

**Figure A3:**
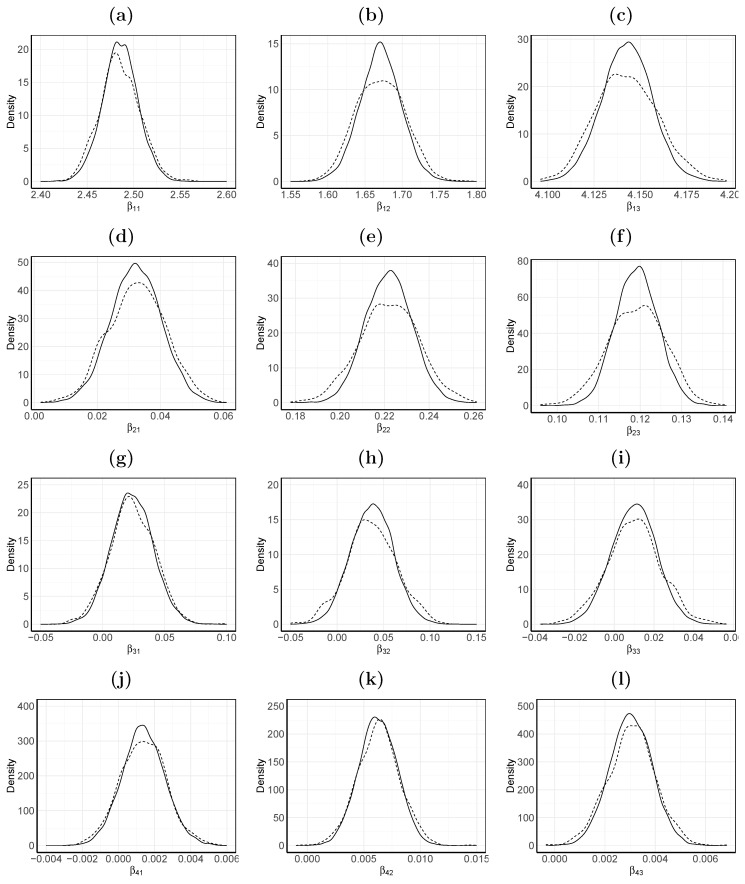
Comparison of the estimated posterior distribution (dashed line) of each regression coefficient with the true posterior distribution (solid line) for a sample size of n=150; log-*t* family.

**Figure A4:**
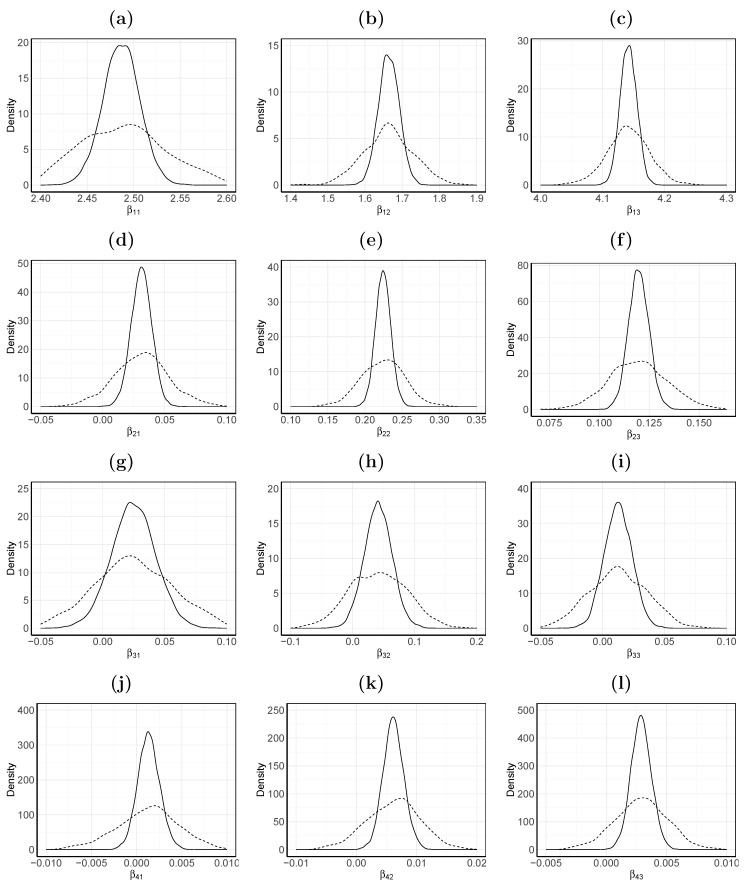
Comparison of the estimated posterior distribution (dashed line) of each regression coefficient with the true posterior distribution (solid line) for a sample size of n=50; log-slash family.

**Figure A5:**
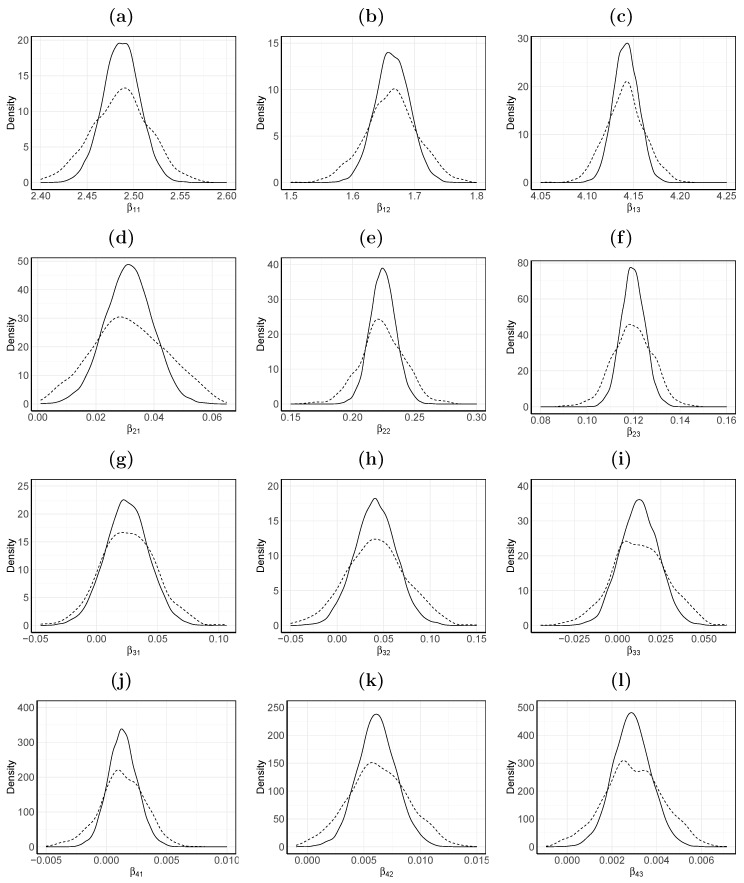
Comparison of the estimated posterior distribution (dashed line) of each regression coefficient with the true posterior distribution (solid line) for a sample size of n=100; log-slash family.

**Figure A6:**
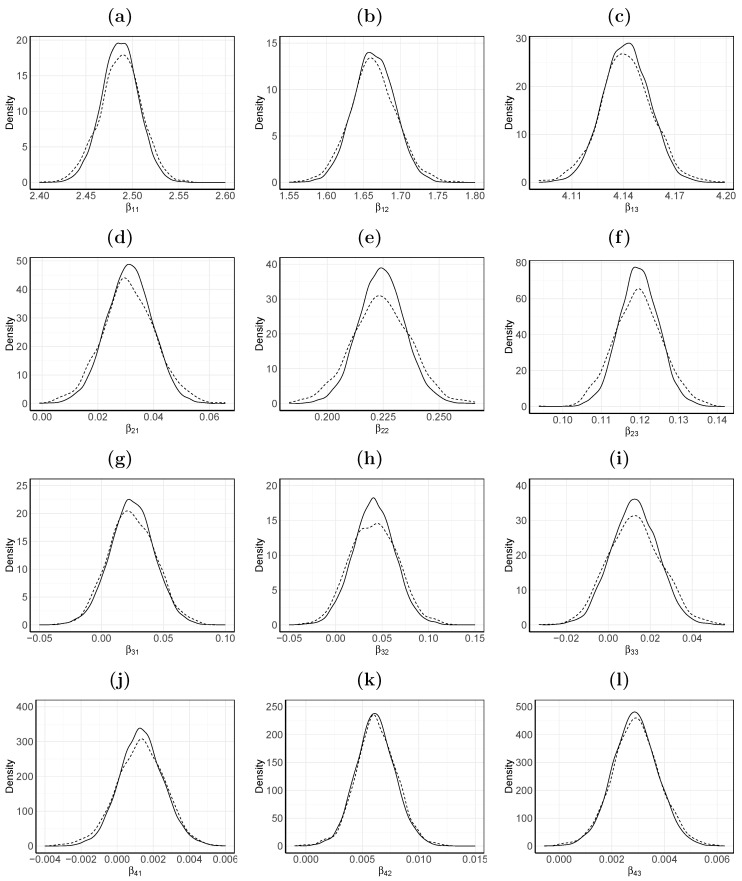
Comparison of the estimated posterior distribution (dashed line) of each regression coefficient with the true posterior distribution (solid line) for a sample size of n=150; log-slash family.

In order to assess the asymptotic behavior of our method, we compare the posterior distributions for each sample size with the true posterior distribution, which we obtain by fitting the corresponding model (log-*t* or log-slash) to the real dataset described in Section 5. Figure A1, Figure A2 and Figure A3 present the estimated posterior distributions of the regression coefficients for the log-*t* family for each sample size, compared with the true posterior distributions. The same comparisons are shown in Figure A4, Figure A5 and Figure A6 for the log-slash case. The plots suggest a satisfactory performance of the estimated posterior distributions. It can be observed that there is increasing agreement between the estimated and true posterior distributions as the sample size increases.

Table 1 presents the median and the median absolute deviation (MAD) of the estimated values for all the parameters associated with the log-*t* and log-slash families, respectively. The medians closely approximate the true parameter values, while the dispersion (MAD) decreases as the sample size grows. The values in these tables indicate that the medians are close to the true parameters and the MAD decreases as the sample size increases, showing satisfactory behavior of the posterior estimators. Let $y_{k,\alpha ,0}$ and $y_{k,\alpha ,1}$, k=1,2,3, 0<α<1, represent the α-quantiles of $Y_{k}$, when $x_{3}=0$ and $x_{3}=1$, respectively. We computed the true quartiles using (12), evaluating the model at the true parameter values and the sample means of the simulated covariates. In Table 2, we report the median and MAD of the estimated values for the quartiles of each response variable. The quantile estimators exhibit satisfactory behavior, as the medians closely approximate the true quartiles, and increasing the sample size yields a lower MAD. Table 3 presents the average execution times of the Bayesian estimation method for each model and sample size. These times include the construction of the monotone missing data pattern and the computation of posterior estimates using 10,000 Monte Carlo replicates. Notably, the execution times for the log-slash model are lower than those for the log-*t* model, primarily due to the weight sampling method (Algorithms 1 and 2). All simulations were performed on a computer equipped with a dual-core Intel Core i3 processor, 8 GB of RAM, and Windows 10 (64-bit), using R [40] (version 4.2.2.). All results presented in this paper can be reproduced using the source code available at https://github.com/joseescobara/MDA-algorithm, accessed on 19 January 2026.

## 5. Application

### 5.1. Description of the Anthropometric Data

Anthropometric growth curves are an essential tool in pediatrics as they assess the adequacy of physiological support for growth and development during early life (World Health Organization [41,42]). Deviations from the pattern described in the growth curves of length/height, weight, head circumference, arm circumference, subscapular skinfold, and triceps skinfold, according to gender and age, may be associated with nutritional disorders and growth abnormalities in infants. The construction of these curves has been made using univariate and multivariate approaches based on complete data (Morán-Vásquez et al. [8,43]). Ignorable missing data are frequent in anthropometric variables (Amugsi et al. [44]), with common causes including measurement or equipment errors, subject non-compliance (e.g., uncooperative children or caregiver refusal), and administrative discrepancies during data entry or processing. The construction of anthropometric growth curves subject to missing observations is challenging, particularly when the goal is to model the association between marginal responses in multivariate data. While many studies discard cases with missing observations (Wei et al. [45], Chang et al. [46], Morán-Vásquez et al. [8]), our model allows for the construction of growth curves for correlated measurements, robust to skewness, outliers, and missing data.

**Figure 1:**
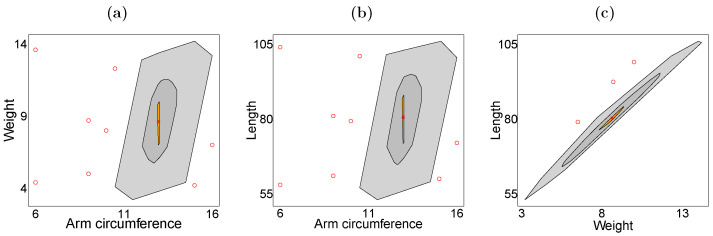
Bagplots of (**a**) weight vs. arm circumference, (**b**) length vs. arm circumference, (**c**) length vs. weight.

**Figure 2:**
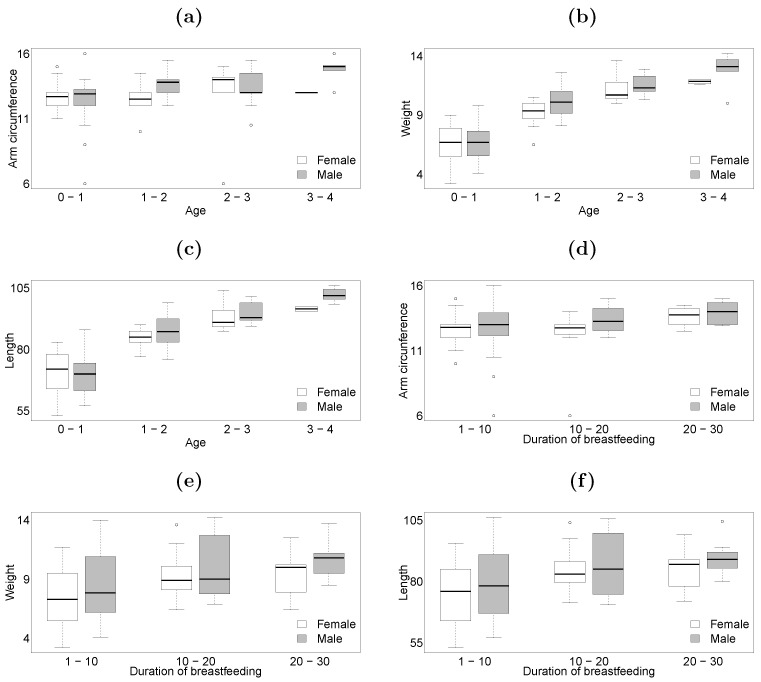
Comparative boxplots by gender (**a**) arm circumference vs. age, (**b**) weight vs. age, (**c**) length vs. age, (**d**) arm circumference vs. duration of breastfeeding, (**e**) weight vs. duration of breastfeeding, (**f**) length vs. duration of breastfeeding; children’s data.

We consider a dataset of children up to and including 4 years old with a confirmed diagnosis of acute malnutrition, available at the website https://medata.gov.co/dataset/1-026-22-000137, accessed on 19 January 2026. The dataset was collected between the years 2016 and 2021 in the Robledo neighborhood, located in the municipality of Medellín, department of Antioquia, Colombia. The trivariate response vector consists of arm circumference (in centimeters), weight (in kilograms), and height (in centimeters), while the covariates are gender (G; 0 for female, 1 for male), age (A; in years), and duration of breastfeeding (B; in weeks). Of the 173 individuals, 8 have missing data for weight, 6 for length, and 63 for arm circumference. The explanatory variables are fully observed. Table 4 presents descriptive statistics for the anthropometric data. The mean and median values indicate a slight skewness in the response variables. The interquartile range suggests moderate variability, consistent with a malnourished child population. With respect to data missingness, arm circumference shows the highest proportion of missing values, whereas weight and length have minimal missing data. The covariates age and breastfeeding duration are fully observed. On the other hand, the bagplots displayed in Figure 1 indicate a positive correlation between arm circumference, weight, and length, with slightly skewed bivariate distributions and the presence of outliers. The comparative boxplots displayed in Figure 2a–f suggest that gender, age, and duration of breastfeeding influence the empirical quantiles of arm circumference, weight, and length.

### 5.2. Results

To investigate the empirical associations involving the anthropometric variables described in Section 5.1, we fitted the multivariate LNI linear regression model (8). The log-slash model offers statistical robustness relative to the log-normal family and outperforms the log-*t* model in computational efficiency (Section 4). Therefore, we consider the trivariate log-slash family, defined where *H* is the CDF of a random variable with the PDF in (4). For the tail parameter, we assume a prior of ν∼Gamma(6,2), as indicated in Section 4.

Table 5 presents the posterior estimates (medians) of the regression coefficients along with the 95% Bayesian credible intervals using equal-tail areas, where the lower and upper bounds are computed as the 2.5% and 97.5% quantiles of the posterior distributions, respectively. The 95% credibility interval for a regression coefficient that contains zero indicates insufficient evidence to conclude that the associated covariate affects the response variable. Conversely, if the interval excludes zero, the covariate is considered to have a credible influence on the response. For example, all credible intervals associated with gender contain zero, indicating no credible effect on any of the response variables. In contrast, all credible intervals associated with age exclude zero, providing strong evidence of a systematic effect. Regarding breastfeeding duration, the credible interval for arm circumference contains zero (indicating no credible effect), whereas there is strong evidence of an effect on weight and length. A one-year increase in age is associated with multiplicative changes in the quantiles of arm circumference, weight, and length by factors of exp(0.03)=1.03, exp(0.22)=1.25, and exp(0.12)=1.13, respectively. In practical terms, this corresponds to estimated increases of 3%, 25%, and 13% for every additional year of age. Analogously, a one-week increase in breastfeeding duration yields multiplicative increases in the quantiles of weight and length (corresponding to percentage increases of 0.6% and 0.3%).

The posterior estimates with the respective 95% Bayesian credible intervals of the other parameters are ${\hat{\psi }}_{11}=0.0050\;\left(0.0033,0.0078\right)$, ${\hat{\psi }}_{22}=0.0128\;\left(0.0097,0.0169\right)$, ${\hat{\psi }}_{33}=0.0032\;\left(0.0024,0.0042\right)$, ${\hat{\psi }}_{12}=0.0019\;\left(0.0002,0.0038\right)$, ${\hat{\psi }}_{13}=0.0010\;\left(0.0002,0.0019\right)$, ${\hat{\psi }}_{23}=0.0058\;\left(0.0044,0.0076\right)$, and $\hat{\nu }=2.2912\;\left(1.6464,3.3711\right)$. Given that ${\hat{\psi }}_{22}>{\hat{\psi }}_{11}>{\hat{\psi }}_{33}$, it is estimated that the weight has the greatest relative dispersion, while the length has the least. The values of ${\hat{\psi }}_{12}$, ${\hat{\psi }}_{13}$, and ${\hat{\psi }}_{23}$ are positive, indicating that arm circumference, weight, and length are associated with one another. Since the value of $\hat{\nu }$ is small, the fit is consistent with a heavy-tailed model.

**Figure 3:**
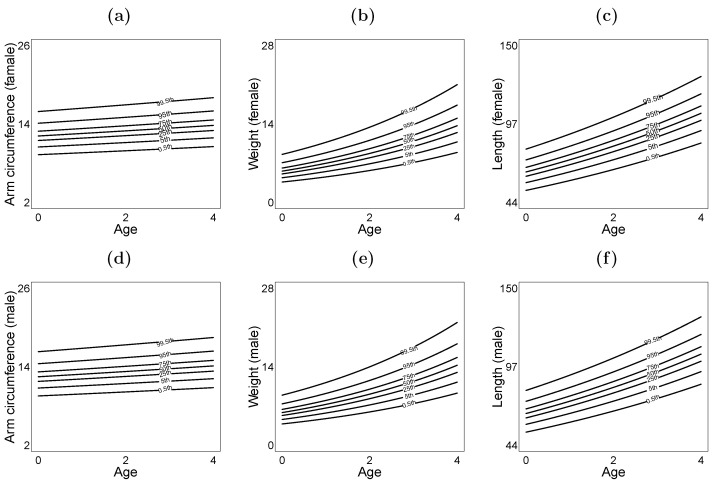
Fitted quantile curves for arm circumference, weight, and length versus age, with breastfeeding duration fixed at the average. (**a**–**c**) Curves for girls. (**d**–**f**) Curves for boys. Displayed curves correspond to the 0.5th, 5th, 25th, 50th, 75th, 95th, and 99.5th percentiles.

Figure 3 illustrates the fitted quantile curves for arm circumference, weight, and length as a function of age. These curves are stratified by gender, with breastfeeding duration held constant at the mean value of 9.18 weeks.

## 6. Discussion and Conclusions

This article implements an MDA algorithm for multivariate data imputation to perform the posterior inference on the parameters of the class of multivariate LNI linear regression models. This approach allows modeling marginal quantiles via a linear regression structure on the median vector parameter, taking into account the association between marginals, potential skewness, possible outliers, and missing data in the response vector. A wide range of regression models can be considered, but we concentrate on the log-*t* and log-slash families, which include an additional parameter that models heavy-tailed data. Simulation studies indicated the satisfactory behavior of the posterior distributions and the quantile estimation technique. Despite the moderate computational cost, execution times of our simulations remain within a manageable range for moderate sample sizes, particularly for multivariate data exhibiting complex characteristics such as incomplete values, skewness, and heavy tails. Applications to children’s anthropometric data demonstrated the practical utility of our methodology. Specifically, the log-slash model effectively captures the multivariate skewness of the data, even in the presence of outliers and missing values. This enabled us to build anthropometric growth curves for arm circumference, weight, and length as a function of age and breastfeeding duration for boys and girls. These curves were obtained by accounting for the association between the response variables and are valuable in pediatrics and nutrition for tracking child development, particularly in the context of malnutrition. All results presented in this paper can be reproduced using the source code available at https://github.com/joseescobara/MDA-algorithm, accessed on 19 January 2026.

Future work will be devoted to developing diagnostic procedures for the models discussed in this paper and extending our methodology to the entire log-elliptical class of distributions (Morán-Vásquez and Ferrari [10]). Future extensions will focus on multivariate regression models with missing data using the log-skew-normal/independent (Morán-Vásquez et al. [47]) and Box-Cox elliptical (Morán-Vásquez and Ferrari [10]) classes of distributions. By incorporating shape parameters, these classes provide a more flexible framework for modeling both multivariate asymmetry and heavy-tailed behavior simultaneously. We also aim to assess the performance of various model selection criteria in this context to effectively choose the most suitable among these competing flexible families.

## Figures and Tables

**Table 1:** Median and MAD of the parameter estimates; log-*t* and log-slash families.

	log-*t*	*n* = 50		*n* = 100		*n* = 150	
	True parameter	Median	MAD	Median	MAD	Median	MAD
$\beta_{11}$	2.4855	2.4839	0.0342	2.4832	0.0206	2.4838	0.0138
$\beta_{21}$	0.0323	0.0324	0.0157	0.0325	0.0080	0.0327	0.0061
$\beta_{31}$	0.0236	0.0234	0.0239	0.0245	0.0150	0.0233	0.0118
$\beta_{41}$	0.0014	0.0014	0.0024	0.0014	0.0012	0.0014	0.0009
$\beta_{12}$	1.6709	1.6689	0.0461	1.6698	0.0290	1.6707	0.0230
$\beta_{22}$	0.2219	0.2229	0.0194	0.2224	0.0118	0.2223	0.0090
$\beta_{32}$	0.0377	0.0384	0.0318	0.0396	0.0209	0.0365	0.0175
$\beta_{42}$	0.0062	0.0060	0.0033	0.0063	0.0019	0.0063	0.0012
$\beta_{13}$	4.1428	4.1410	0.0236	4.1425	0.0150	4.1423	0.0112
$\beta_{23}$	0.1192	0.1192	0.0096	0.1194	0.0058	0.1193	0.0047
$\beta_{33}$	0.0101	0.0105	0.0152	0.0105	0.0103	0.0101	0.0085
$\beta_{43}$	0.0030	0.0029	0.0016	0.0031	0.0009	0.0031	0.0006
$\psi_{11}$	0.0066	0.0100	0.0022	0.0075	0.0014	0.0070	0.0011
$\psi_{22}$	0.0169	0.0243	0.0043	0.0204	0.0031	0.0199	0.0024
$\psi_{33}$	0.0042	0.0061	0.0011	0.0052	0.0008	0.0049	0.0006
$\psi_{12}$	0.0027	0.0033	0.0023	0.0029	0.0014	0.0028	0.0010
$\psi_{13}$	0.0014	0.0017	0.0011	0.0015	0.0007	0.0015	0.0005
$\psi_{23}$	0.0077	0.0105	0.0020	0.0089	0.0014	0.0086	0.0011
ν	5.6875	10.3844	1.3688	6.3750	1.4750	5.5125	0.8844
	log-slash	n=50		n=100		n=150	
	True parameter	Median	MAD	Median	MAD	Median	MAD
$\beta_{11}$	2.4867	2.4874	0.0339	2.4868	0.0207	2.4880	0.0144
$\beta_{21}$	0.0313	0.0322	0.0140	0.0309	0.0089	0.0308	0.0062
$\beta_{31}$	0.0247	0.0243	0.0224	0.0258	0.0153	0.0240	0.0132
$\beta_{41}$	0.0013	0.0016	0.0022	0.0013	0.0012	0.0013	0.0009
$\beta_{12}$	1.6639	1.6618	0.0439	1.6634	0.0263	1.6623	0.0196
$\beta_{22}$	0.2243	0.2249	0.0195	0.2235	0.0113	0.2240	0.0087
$\beta_{32}$	0.0411	0.0402	0.0331	0.0417	0.0214	0.0416	0.0177
$\beta_{42}$	0.0061	0.0064	0.0029	0.0062	0.0017	0.0062	0.0012
$\beta_{13}$	4.1417	4.1411	0.0219	4.1410	0.0135	4.1414	0.0097
$\beta_{23}$	0.1197	0.1192	0.0097	0.1197	0.0057	0.1195	0.0043
$\beta_{33}$	0.0125	0.0124	0.0164	0.0123	0.0107	0.0127	0.0087
$\beta_{43}$	0.0029	0.0029	0.0014	0.0029	0.0009	0.0029	0.0006
$\psi_{11}$	0.0050	0.0066	0.0019	0.0057	0.0012	0.0052	0.0008
$\psi_{22}$	0.0128	0.0166	0.0036	0.0152	0.0022	0.0146	0.0018
$\psi_{33}$	0.0032	0.0042	0.0009	0.0038	0.0006	0.0036	0.0005
$\psi_{12}$	0.0019	0.0022	0.0014	0.0021	0.0011	0.0021	0.0008
$\psi_{13}$	0.0010	0.0010	0.0008	0.0011	0.0050	0.0011	0.0004
$\psi_{23}$	0.0058	0.0072	0.0017	0.0066	0.0010	0.0063	0.0004
ν	2.2912	2.6129	0.4351	2.4672	0.3356	2.3307	0.2392

**Table 2:** Median and MAD of estimated quartiles; log-*t* and log-slash families.

	log-*t*	*n* = 50		*n* = 100		*n* = 150	
	True quartile	Median	MAD	Median	MAD	Median	MAD
$y_{1,1/4,0}$	12.098	11.882	0.2396	12.008	0.1564	12.034	0.1196
$y_{1,1/2,0}$	12.828	12.724	0.2313	12.781	0.1534	12.790	0.1103
$y_{1,3/4,0}$	13.602	13.641	0.2703	13.603	0.1811	13.581	0.1366
$y_{2,1/4,0}$	7.4103	6.9451	0.1823	7.1717	0.1338	7.1817	0.1042
$y_{2,1/2,0}$	8.1381	7.7472	0.1885	7.9470	0.1309	7.9444	0.1127
$y_{2,3/4,0}$	8.9372	8.6509	0.2342	8.8027	0.1596	8.7913	0.1361
$y_{3,1/4,0}$	75.310	72.805	0.9149	74.056	0.6702	74.048	0.5686
$y_{3,1/2,0}$	78.928	76.941	0.9059	77.961	0.6602	77.905	0.5433
$y_{3,3/4,0}$	82.719	81.235	1.0604	82.108	0.7394	81.955	0.6286
$y_{1,1/4,1}$	12.387	12.158	0.2017	12.300	0.1352	12.322	0.1025
$y_{1,1/2,1}$	13.134	13.030	0.1753	13.095	0.1246	13.091	0.0925
$y_{1,3/4,1}$	13.927	13.953	0.2287	13.932	0.1504	13.908	0.1184
$y_{2,1/4,1}$	7.6952	7.2327	0.1574	7.4548	0.1076	7.4514	0.0969
$y_{2,1/2,1}$	8.4509	8.0552	0.1503	8.2624	0.1153	8.2531	0.0929
$y_{2,3/4,1}$	9.2808	8.9783	0.1906	9.1633	0.1380	9.1356	0.1140
$y_{3,1/4,1}$	76.077	73.632	0.7480	74.829	0.5295	74.807	0.4844
$y_{3,1/2,1}$	79.731	77.795	0.7679	78.792	0.4992	78.721	0.4337
$y_{3,3/4,1}$	83.561	82.199	0.9032	82.952	0.6092	82.815	0.5120
	log-slash	n=50		n=100		n=150	
	True quartile	Median	MAD	Median	MAD	Median	MAD
$y_{1,1/4,0}$	12.090	11.900	0.2381	12.002	0.1556	12.040	0.1299
$y_{1,1/2,0}$	12.816	12.720	0.2209	12.759	0.1423	12.775	0.1294
$y_{1,3/4,0}$	13.587	13.580	0.2569	13.556	0.1674	13.556	0.1428
$y_{2,1/4,0}$	7.3833	6.9616	0.1798	7.1578	0.1366	7.1699	0.1036
$y_{2,1/2,0}$	8.1051	7.7225	0.1856	7.9108	0.1368	7.9107	0.1091
$y_{2,3/4,0}$	8.8975	8.5728	0.2212	8.7549	0.1577	8.7353	0.1326
$y_{3,1/4,0}$	75.206	72.989	0.9343	73.992	0.6831	74.037	0.5483
$y_{3,1/2,0}$	78.793	76.883	0.9490	77.829	0.6775	77.794	0.5484
$y_{3,3/4,0}$	82.551	81.052	1.0388	81.853	0.7553	81.761	0.5811
$y_{1,1/4,1}$	12.393	12.222	0.2155	12.319	0.1368	12.337	0.1070
$y_{1,1/2,1}$	13.137	13.038	0.1919	13.096	0.1215	13.096	0.1060
$y_{1,3/4,1}$	13.927	13.918	0.2265	13.916	0.1450	13.901	0.1315
$y_{2,1/4,1}$	7.6932	7.2529	0.1526	7.4608	0.1116	7.4643	0.0843
$y_{2,1/2,1}$	8.4453	8.0502	0.1583	8.2478	0.1090	8.2410	0.0880
$y_{2,3/4,1}$	9.2710	8.9323	0.1852	9.1225	0.1410	9.1050	0.1116
$y_{3,1/4,1}$	76.151	73.837	0.8078	74.985	0.5513	74.952	0.4233
$y_{3,1/2,1}$	79.783	77.819	0.7423	78.828	0.5426	78.778	0.4116
$y_{3,3/4,1}$	83.588	81.975	0.8671	82.899	0.6530	82.766	0.4985

**Table 3:** Average execution time (in minutes) for each family.

Family	n=50	n=100	n=150
log-*t*	5.05	8.40	11.9
log-slash	3.36	5.23	8.21

**Table 4:** Mean, median, standard deviation (SD), interquartile range (IQR), and percentage of missing values of anthropometric variables by gender.

Gender	Variable	Mean	Median	SD	IQR	Missing (%)
Female	Arm circumference (cm)	12.6	13.0	1.48	1.00	38.8
	Weight (kg)	8.23	8.15	2.28	3.48	7.46
	Length (cm)	79.2	80.0	11.1	15.7	4.48
	Age (years)	1.61	1.00	1.07	1.08	0.00
	Breastfeeding (weeks)	9.72	8.00	6.49	9.50	0.00
Male	Arm circumference (cm)	13.1	13.0	1.61	1.70	34.9
	Weight (kg)	8.89	9.00	2.67	4.30	2.83
	Length (cm)	81.2	81.0	13.6	22.7	2.83
	Age (years)	1.68	1.00	1.16	2.00	0.00
	Breastfeeding (weeks)	8.85	6.00	7.04	8.00	0.00

**Table 5:** Estimated posterior medians and 95% credibility intervals for the regression coefficients; children’s data.

Response Variable	Explanatory Variable	Estimate	Lower	Upper
Arm circumference	Intercept	2.4867	2.4473	2.5253
	Age	0.0313	0.0150	0.0470
	Gender	0.0247	−0.0103	0.0594
	Breastfeeding	0.0013	−0.0010	0.0038
Weight	Intercept	1.6639	1.6097	1.7183
	Age	0.2243	0.2041	0.2451
	Gender	0.0411	−0.0028	0.0855
	Breastfeeding	0.0061	0.0028	0.0097
Length	Intercept	4.1417	4.1159	4.1677
	Age	0.1197	0.1097	0.1300
	Gender	0.0125	−0.0087	0.0342
	Breastfeeding	0.0029	0.0012	0.0046

## Data Availability

The code and data required to reproduce the results presented in this article are available at https://github.com/joseescobara/MDA-algorithm, accessed on 19 January 2026.
